# Impact of Transgenic *Cry1Ab/2Aj* Maize on Abundance of Non-Target Arthropods in the Field

**DOI:** 10.3390/plants11192520

**Published:** 2022-09-26

**Authors:** Yan Yang, Yi Chen, Jiabao Xue, Yuanyuan Wang, Xinyuan Song, Yunhe Li

**Affiliations:** 1 Key Laboratory of Genetics and Germplasm Innovation of Tropical Special Forest Trees and Ornamental Plants, Ministry of Education, College of Forestry, Hainan University, Haikou 570228, China; 2 State Key Laboratory for Plant Diseases and Insect Pests, Institute of Plant Protection, Chinese Academy of Agricultural Sciences, Beijing 100193, China; 3 Institute of Tropical Bioscience and Biotechnology, Chinese Academy of Tropical Agricultural Sciences, Haikou 571025, China; 4 Sanya Research Institute of the Chinese Academy of Tropical Agricultural Sciences, Sanya 572022, China; 5 Hainan Yazhou Bay Seed Laboratory, Sanya 572025, China; 6 Agro-Biotechnology Research Institute, Jilin Academy of Agricultural Sciences, Changchun 130033, China

**Keywords:** *Bacillus thuringiensis*, non-target arthropods, biodiversity, community composition, ecological safety assessment

## Abstract

Transgenic *Bacillus thuringiensis* (*Bt*) maize has broad prospects for application in China. Before commercialization, it is necessary to assess possible ecological impacts, including impacts on non-target arthropods (NTAs) in the field. In the present study, transgenic *Bt* maize expressing *cry1Ab/2Aj* and its corresponding non-transformed near isoline were planted under the same environmental and agricultural conditions, and arthropods in the field were collected during the three main growth stages of maize. In a one year trial, the results showed the composition of NTA communities in the transgenic and control maize fields were similar. There were no significant differences for community-level parameters of species richness (*S*), Shannon–Wiener diversity index (*H′*), evenness index (*J*) and Simpson’s dominant concentration (*C*) between the two types of maize fields. Likewise, a Bray–Curtis dissimilarity and distance analysis showed that Cry1Ab/2Aj toxin exposure did not increase community dissimilarities between *Bt* and non-*Bt* maize plots and that the structure of the NTAs community was similar on the two maize varieties. Furthermore, planting of the transgenic *cry1Ab/2Aj* maize did not affect the density or composition of non-target decomposers, herbivores, predators, parasitoids and pollinator guilds. In summary, our results showed that planting of *Bt* maize producing Cry1Ab/Cry2Aj proteins do not adversely affect population dynamics and diversity of NTAs.

## 1. Introduction

The past 23-year planting history (1996–2018) of genetically engineered (GE) crops have demonstrated the socioeconomic benefits of genetically engineered (GE) crops, including: (1) increased productivity and global food and feed security; (2) support for self-sufficiency in a country’s arable land; (3) protect biodiversity, prevent deforestation, and protect biodiversity reserves; (4) mitigate challenges related to climate change; and (5) improve economic, health, and social benefits [1,2]. The global area of GM crops has reached 190.4 million hectares in 2019, including over 29 countries [3]. Among them, GE maize is the third most important crop in terms of GE crop area and has reached 31% of the global maize crop area. With more than 24 years of GE maize commercialization in above ten foreign countries, the planting area reached 60.9 million hectares of GE maize in 2019 [3]. In China several GE maize lines expressing insect-resistant or/and herbicide-resistant traits has obtained biosafety certificates, and they will be approved for agricultural production in the near future [4,5]. China is the second largest maize producer and consumer in the world, with a large cultivated area and distribution throughout the whole country. Maize is not only the staple food crop, but also an important raw material for feed and industry. If GE maize is introduced and planted on a large scale, it may change the ecological environment of the original field and the living environment of pests, thus causing a series of safety problems and non-target effects [4].

The impact of *Bt* crops on non-target arthropods (NTAs) is an important part of an ecological risk assessment. Laboratory studies have been extensively conducted to evaluate the impact of *Bt* crops on NTAs, focusing the functional groups that play important ecological roles in farmland systems, such as natural enemies, pollinators, and economically important insects [6,7,8,9,10,11]. Systematic analyses of these research data found that the Bt insecticidal proteins produced by GE crops have a very narrow insecticidal spectrum, strong insecticidal specificity and generally have no negative effects on non-target arthropods [11,12,13,14]. In addition to carrying out bioassay experiments under controlled conditions in laboratories, it is generally required to further conduct field experiments to evaluate whether the planting of GE crops affects the population structure and density of NTAs [15]. Field experiments were conducted to investigate whether there are differences in NTA species, densities and genotypes when GE crops are grown in the field compared with non-GE crops. Such short-term field investigation conducted before the commercialization of GE crops normally show no effects on arthropod populations [15,16,17,18]. However, a number of long-term and large-scale monitoring studies conducted after the commercialization of a GE crop do indicate that planting of *Bt* crops can change the arthropod population structure and dynamics in the field, for example, the increase of certain secondary insect pest populations [19,20,21]. These effects were widely considered to be caused by the change of farmland practices, such as the reduced application of pesticides associated with reduction of target pest populations. For example, transgenic insect-resistant cotton can effectively control cotton bollworm (*Helicoverpa armigera*); therefore, the use of pesticides has been greatly reduced for controlling this pest, which indirectly results in the break of the non-target pest mirid bug [22].

NTA effects need to be conducted on a case-by-case basis using a weight of evidence approach and considering all relevant information [23]. In recent years, China has developed a number of GE maize lines exhibiting high efficacy in target pest control that have to undergo a strict risk assessment before going to commercial use [24]. A transgenic maize line Shuangkang 12-5 (SK12-5), developed through the *Agrobacterium*-mediated method [25,26] that expresses a *cry1Ab/2Aj* fusion gene and an *EPSPS* gene [27], showed efficient field resistance against lepidopteran pests *Ostrinia furnacalis* (Guenée) and *Helicoverpa armigera* (Hübner) and high herbicide tolerance to glyphosate [26]. The GE maize line has already passed regulatory approval and received a safety certification, and it may enter commercial cultivation soon in China. So far, studies have been conducted with the maize line, mainly focusing on the development of detection methods [28,29], resistance on target pests [26,30] and laboratory assessment of the potential effects on non-target arthropods, including honey bee (*Apis mellifera*) [31], silkworm (*Bombyx mori*) [32], green lacewing (*Chrysoperla sinica*) [33] and effects on microbial diversity [34]. Its potential effects on arthropod populations have rarely been evaluated in the field.

In the present study, the transgenic *cry1Ab/2Aj* maize Shuangkang 12-5 (SK12-5) and its corresponding non-transformed near isoline Lianchuang 303 (LC303) [27] were tested. Number of species, abundance and population characteristics of NTAs collected during three main growth periods of maize were compared between the two types of maize fields, and the species diversity of the arthropod community were further calculated and analyzed. The results will complement knowledge of the impact of transgenic *Bt* maize on the safety of NTAs in the field and will provide a reference for decision-making on commercial application of the SK12-5 maize line in China and maybe in other countries.

## 2. Results

### 2.1. Species Composition of NTAs in Different Maize Fields

**Figure 1:**
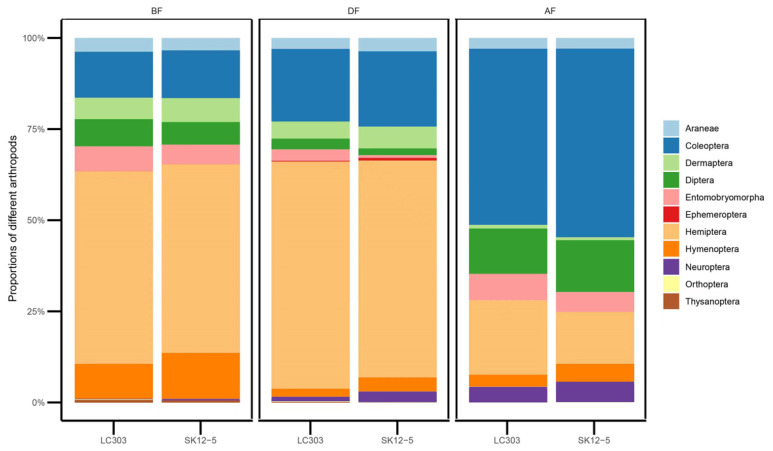
Proportions of all orders of non-target arthropods (NTAs) found in SK12-5 (*Bt*) and LC303 (non-*Bt*) maize fields in three main maize growth stages: BF—Before Flowering stage; DF—During Flowering stage; AF—After Flowering stage.

During the BF (before flowering) stage, 45 species of NTAs belonging to 11 orders and 38 families were detected in non-*Bt* maize fields, and 53 species belonging to 10 orders and 41 families were detected in *Bt* maize fields. There were 39 species of NTAs detected simultaneously in both *Bt* and non-*Bt* maize fields (Table 1), and the Czekanowski Community Similarity Coefficient (*C_S_*) of the species composition of the two communities was 0.73 (Table 2). In both *Bt* and non-*Bt* maize fields, Hemiptera and Coleoptera were the dominant orders, accounting for 51.7 % and 52.8 %, and 13.1 % and 12.6 %, in transgenic and non-transgenic maize, respectively (Figure 1). The compositions of NTAs in *Bt* and non-*Bt* maize fields were not significantly different (Student’s *t*-test, all *p* > 0.05).

In the DF (during flowering) stage, the total number of species detected in non-*Bt* maize fields was 47, belonging to 11 orders and 35 families, and in *Bt* maize fields there were 50 species belonging to 10 orders and 39 families. The number of commonly detected species in both *Bt* and non-*Bt* maize fields was 37 (Table 1), and the *C_S_* of the species composition of the two communities was 0.67 (Table 2). In both *Bt* and non-*Bt* maize fields, Hemiptera and Coleoptera were the dominant orders, accounting for 59.5 % and 62.3 %, and 20.7 % and 19.9 %, in transgenic and non-transgenic maize, respectively (Figure 1). NTAs in the order Neuroptera were significantly greater in *Bt* maize fields than in non-*Bt* maize fields (Student’s t-test, *F* = 36.84, *p* = 0.009); the composition of other NTAs in *Bt* and non-*Bt* maize fields were not significantly different (Student’s *t*-test, all *p* > 0.05). In the AF (after flowering) stage, there were 36 species detected in non-*Bt* maize fields, belonging to 8 orders and 27 families; 51 species were detected in *Bt* maize fields, belonging to 9 orders and 36 families. The number of species detected in both fields were 33 (Table 1), and the *C_S_* of the two communities was 0.70 (Table 2). Further, in both *Bt* and non-*Bt* maize fields, Coleoptera and Hemiptera were the dominant orders, accounting for 51.8 % and 48.3 %, and 14.2 % and 20.4 %, in transgenic and non-transgenic maize, respectively (Figure 1). There were no significant differences in the composition of arthropod species between *Bt* and non-*Bt* maize fields (Student’s *t*-test, all *p* > 0.05).

### 2.2. Community Parameters of NTAs in Different Maize Fields

**Figure 2:**
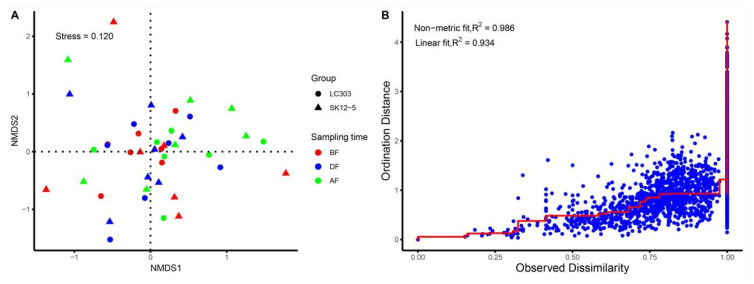
(**A**) Nonmetric Multidimensional Scaling (NMDS based on Bray–Curtis distance) plot of community structures of arthropods for three main growth stages in SK12-5 and LC303 maize fields. Triangles indicate SK12-5 (*Bt*) maize fields, and circles indicate LC303 (non-*Bt*) maize fields. Different colors indicate the three main growth stages: red indicates Before Flowering (BF) stage, blue indicates During Flowering (DF) stage and green indicates After Flowering (AF) stage. (**B**) NMDS Shepard plot.

The NTA community structures in *Bt* and non-*Bt* maize fields were further explored using non-metric multidimensional scaling analysis (NMDS). The distance was estimated using the Bray–Curtis dissimilarity index. Differences in the composition of the NTAs community in the three main maize growth stages were visualized in an NMDS plot (Figure 2). No differences of NTA community structures among *Bt* and non-*Bt* maize fields were observed.

The species richness (*S*), Shannon–Wiener diversity index (*H′*) and evenness (*J*) indexes of NTAs in *Bt* maize fields in the BF, DF and AF maize growth stages were all higher than those in non-*Bt* maize fields, while the Simpson’s dominant concentration (*C*) in *Bt* maize fields were all lower than those in non-*Bt* maize fields (Table 2). However, the Simpson’s dominant concentration (*C*) between *Bt* and non-*Bt* maize fields differed significantly in the BF stage (Student’s *t*-test, *F* = 11.99, *p* = 0.005); differences in other parameters between *Bt* and non-*Bt* maize fields were not significant (Student’s *t*-test, all *p* > 0.05).

### 2.3. Dynamic Comparison of Composition and Community of NTAs at Different Time

In total, the numbers of NTA species in the DF stage were the highest and in the AF stage were the lowest among the three growth stages for both *Bt* and non-*Bt* maize fields; however, there were no significant differences for the three growth stages in both *Bt* and non-*Bt* maize fields (one-way ANOVA followed by Tukey HSD test or Mann–Whitney *U*-test, all *p* > 0.05).

For NTA community parameters (Table 2), the highest species richness (*S*) was in the DF stage for non-*Bt* maize fields and in the BF stage for *Bt* maize fields; the lowest *S* values for both *Bt* and non-*Bt* maize fields were in the AF stage. For the Shannon–Wiener diversity index (*H′*), the highest values for both the *Bt* and non-*Bt* maize fields were in the DF stage, and the lowest for non-*Bt* maize fields in the BF stage and for *Bt* maize fields in the AF stage. The highest evenness index (*J*) was in the BF stage for non-*Bt* maize fields and in the DF stage for *Bt* maize fields, and the lowest were in the AF stage for both *Bt* and non-*Bt* maize fields. For Simpson’s dominant concentration (*C*), the highest value for non-*Bt* maize fields was in the AF stage and for *Bt* maize fields in the BF stage, and the lowest for both *Bt* and non-*Bt* maize fields in the DF stage. There were no significant differences for the three growth stages among *Bt* and non-*Bt* maize fields (one-way ANOVA followed by Tukey HSD test, all *p* > 0.05).

### 2.4. Communities Similarity

In the case of multiple surveys and a large number of species, the NMDS model can more accurately reflect the numerical sort of information of the distance matrix. Thus, the similarities of NTAs community composition across maize type and sampling time were visualized using an NMDS based on a Bray–Curtis dissimilarity matrix. A stress function ranged from 0 to 1 was used to assess the goodness of fit between the ordination and the original data of NTAs. The stress values were 0.12, which suggested that the ordination accurately represented the dissimilarity between samples (Figure 2A). A Shepard diagram of non-metric fit illustrated that observed dissimilarities and the ordination distances were highly correlated (non-metric fit was 0.986) (Figure 2B). The samples collected in the NMDS plot were not separated by sampling time and maize type (Figure 2A,B), which was confirmed by a more detailed analysis of similarity (ANOSIM). No significant correlation was detected between arthropod community composition and sampling time (R^2^ = 0.023, *p* = 0.74). No significant correlation was detected between arthropod community composition and maize type (R^2^ = 0.0033, *p* = 0.45).

### 2.5. Ecosystem Functioning Composition of NTA Communities in Different Maize Fields

**Figure 3:**
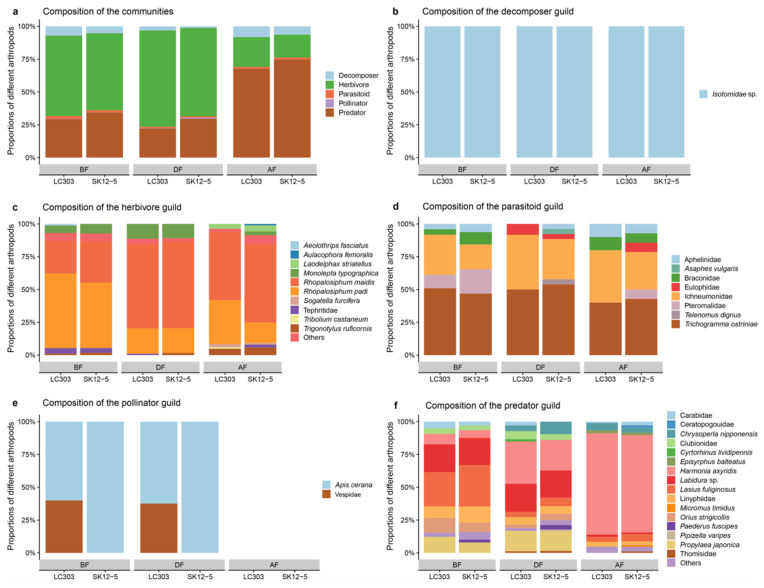
Composition of non-target arthropod (NTA) communities in SK12-5 (*Bt*) and LC303 (non-*Bt*) maize fields for three main maize growth stages: BF—Before Flowering, DF—During Flowering and AF—After Flowering. (**a**) NTA communities; (**b**) decomposers; (**c**) herbivores; (**d**) parasitoids; (**e**) pollinators; and (**f**) predators.

Five functional guilds were identified in *Bt* and non-*Bt* maize fields during the study period. The results showed that for the BF and DF stages, the most abundant guilds in *Bt* and non-*Bt* maize fields were herbivores, followed by predators, decomposers, parasitoids and pollinators. For the AF stage, the most abundant guilds in *Bt* and non-*Bt* maize fields were predators, followed by herbivores, decomposers and parasitoids. The number of pollinators increased in the DF stage and was reduced to zero in the AF stage, comprising a rare guild (Figure 3a,e). As decomposers, only *Isotomidae* sp. was observed in the three main maize growth stages (Figure 3b). For herbivores, Aphididae was the most abundant family, although the common species detected were not exactly the same among different stages; *Rhopalosiphum padi* and *R. maidis* were the most abundant aphids in three main growth stages (Figure 3c). For parasitoids, *Trichogramma ostriniae* and Family Ichneumonidae were the abundant groups for different stages (Figure 3d). For predators, the non-*Bt* maize field had eight common groups at the BF stage: *Lasius fuliginosus*, *Labidura* sp. *Propylaea japonica, Harmonia axyridis*, *Orius strigicollis,* Linyphiidae, Carabidae and Clubionidae, respectively, and the *Bt* maize field had nine common groups—the extra one was *Paederus fuscipes.* At the DF stage, the number of common groups was higher than at the BF stage, *H. axyridis*, *Labidura* sp. and *P. japonica* were the most abundant groups. At the AF stage, *H. axyridis* was the obvious dominant species (Figure 3f). During the entire study period, the composition of NTA communities was essentially uniform among *Bt* and non-*Bt* maize fields.

## 3. Discussion

Transgenic *Bt* crops can bring great advantages for reducing pesticide usage, increasing crop yields and increasing farm income [35,36]. On the other hand, planting of insect-resistant *Bt* crops may pose negative effects on the environment. It is therefore necessary to assess the potential environmental effects of *Bt* crops before being commercialized, including field surveys to evaluate their potential effects on the population dynamics of NTAs [15].

In the current study, SK12-5 transgenic *Cry1Ab/2Aj* maize was selected to assess its potential effects on the composition and community structure of NTAs since it exhibited high efficacy in controlling target pests and it may be commercially planted in China in the near future. The NTA populations were investigated during three main growth stages of maize, that is, the before flowering (BF), during flowering (DF) and after flowering (AF) stages separately. The main reasons for selecting these three periods are that: (1) the highest Cry1Ab/2Aj protein content is expressed in the pollen (data not published) and (2) beneficial insects begin to increase during the maize silking period [37].

During the three maize stages, no non-target lepidopteran species were found in both *Bt* and non-*Bt* fields. For target Lepidopteran species, such as *O. furnacalis,* the total number of the insects captured was only four in *Bt* maize field, and therefore, the lepidopteran insects were not involved for analysis. The results in this study showed no significant differences on the composition, species richness (*S*), Shannon–Wiener diversity index (*H′*), evenness index (*J*), Simpson’s dominant concentration (*C*), community similarity and ecosystem functioning composition of the NTAs community between transgenic insect-resistant maize (SK12-5 transgenic *Cry1Ab/2Aj*) and the non-GE maize. These results are consistent with previous studies showing that *Bt* maize producing Cry1Ab [18,38,39,40,41,42,43,44], Cry1Ab and Cry2Ab [45], Cry1A.105 and Cry2Ab [46,47], Cry1Ac [48,49,50], Cry1A.105 and Cry2Ab and Cry3Bb [51], Cry1Ab and Cry1Ac [52], Cry1Ab and VIP3A [53], Cry1F [54,55,56], Cry1Ah [57], Cry1Ie [58,59,60], Cry34Ab1 and Cry35Ab1 [55,61], Cry34Ab1 and Cry35Ab1 and Cry1F [55,61], Cry3Bb [62,63,64,65] and VIP 3A [66] insecticidal proteins did not affect populations of NTAs in the field. Similarly, there were no significant effects detected in the majority of such filed experiments with *Bt* cotton and *Bt* rice [37,67,68,69]. However, there are indeed studies reporting that cultivation of *Bt* crops can alter population structure and dynamics of non-target arthropods in the field [70]. For example, many studies show that growing of *Bt* crops significantly reduces the density of parasitoids that are specific to the target pests of the *Bt* crops, and the reduction is associated with the decrease of target pest populations [11]. In addition, it has also been frequently reported that the long-term growing of *Bt* crops leads to population increases of non-target pest species, for example, the cultivation of *Bt* cotton in China causes a rise of the secondary non-target pest mirid bugs to become the major pest [22]. The same ecological phenomenon was also found for aphids in *Bt* maize fields [71]. The secondary insect pest population increase has been confirmed to be the consequence that they are not susceptible to or have decreased susceptibility to the *Bt* proteins, and such species would have been controlled by the insecticides that were used for controlling target pests before the commercial use of the *Bt* crops [71]. Meanwhile, studies showed that the cultivation of transgenic insect-resistant crops could protect or improve arthropod biodiversity with the reduced use of pesticides in the field [64,72,73]. This information demonstrates that the current data with the transgenic maize SK12-5 showing no negative impact on the number and community structure of NTAs are valuable for decision-making for commercialization of the *Bt* maize line, but it is a short-term field investigation in small-scale field plots that cannot represent the long-term monitoring on large-scale farms, which should be conducted after commercial planting of *Bt* crops.

The current results showed that Hemiptera was the dominant order and aphids were the dominant species at the BF and DF stages, while at the AF stage, Coleoptera was the dominant order and *H*. *axyridis* were the dominant species. This ecological niche change may be attributed to the tritrophic interaction of aphids and ladybirds. There are micro-balances in nature, where species restrict and counter-restrict each other. The number of aphids increasing in the field will subsequently lead to the increase of the ladybug population since aphids are dominant preys of ladybirds [74]. In reverse, the growth of the aphid population will be inhibited by the increased ladybird population, which belongs to the negative feedback regulation in the biological community [75]. Notably, the same negative ecosystem feedback occurred in both *Bt* and non-*Bt* maize fields, illustrating that the cultivation of transgenic *Cry1Ab/2Aj* maize had a similar impact on the ecosystem with the conventional non-transgenic maize. Besides, *Apis cerana* was the dominant pollinator in *Bt* and non-*Bt* maize fields, but Vespidae were not observed in *Bt* field, which may be due to few lepidopteran pests in *Bt* fields, considering lepidopteran pests are the main preys of Vespidae species. Overall, the results showed that composition of NTA species between the *Bt* and non-*Bt* maize fields was similar. By comparing and analyzing the structural dynamics and similarities of the community, the degree of similarity between different communities can be more objectively reflected. *C_S_*, referring to the similarity of species composition between communities, between the *Bt* and non-*Bt* maize fields for the three growth stages were all greater than 0.5, showing that the degree of similarity between communities is high [76]. At the community level, there were no significant differences in the three growth stages in terms of overall analysis of species richness (*S*), Shannon–Wiener diversity index (*H′*), evenness index (*J*) and Simpson’s dominant concentration (*C*) of the NTA community between *Bt* and non-*Bt* maize fields. The temporal trend for each parameter was consistent, and there were no significant differences. The results indicate that transgenic *Cry1Ab/2Aj* maize SK12-5 has no obvious effect on the composition and community parameters of NTAs.

In recent years, NMDS analysis methods have been used for many types of ecological studies and evaluating the impact of transgenic crop cultivation on animal communities in the field [59,60,77]. This study also used this method to analyze the relationship between maize type and NTAs composition and showed that transgenic maize did not have a significant impact on NTAs. The analysis of NTA communities examined at the species and family levels demonstrated that the composition of the dominant, common and rare guilds or species and families was similar in *Bt* and non-*Bt* maize fields. Compared with populations, biological communities have higher structure and more complex diversity. Results of this study show that at the community level, insect-resistant *Cry1Ab/2Aj* maize had no significant ecological impacts on the NTAs community in the field. This finding complements the knowledge of potential effects of insect-resistant GE crops on arthropod populations and provides valuable information for decision-making on commercial application of the *Bt* maize line SK12-5 in China.

## 4. Material and Methods

### 4.1. Field Planting and Management Methods

Transgenic *cry1Ab/2Aj* maize Shuangkang 12-5 (SK12-5) and its corresponding non-transformed near-isoline Lianchuang 303 (LC303) seeds were sown simultaneously in experimental fields in the Jilin Academy of Agricultural Sciences, Gongzhuling City, Jilin Province, China (43°19′ N, 124°29′ W). These maize lines were grown in adjacent (50 m^2^) plots, with three replicate plots for each maize line. All maize plants were cultivated equally according to the common local agricultural practices in 2018; no chemical pesticides and herbicides were applied throughout the growing season of maize, and other farming operations were the same as the local routine. Thus, the two maize lines were grown under the same environmental and agricultural conditions.

### 4.2. Sample Collection and Identification

NTAs were sampled for 7 days monthly in three maize growth stages: before flowering stage (abbreviated BF), during flowering stage (abbreviated DF) and after flowering stage (abbreviated AF). Two sampling methods were used to collect NTAs in maize fields—direct sampling and trapping: (1) direct sampling included visual observations to capture taxa occurring on maize plants and sweep nets to capture aerial taxa. In each plot, visual inspections of plants were made row after row to collect all arthropods found every day, and a sweep net was used to capture flying arthropods every day. Sampling was conducted in the morning when arthropods were less active. (2) Trapping sampling used pitfall traps to capture surface- and ground-dwelling taxa. Five pitfall traps (a plastic cup of 11-cm depth half-filled with water and scouring agent) were established in an “X” pattern that covered the whole plot, regularly distributed over the plot length but at least 2 m from the field border. Pitfall traps were set for 24 h, and the trapping agent was changed after gathering the collected arthropods every day.

The collected arthropods were placed separately into centrifuge tubes and immediately frozen at −20 °C in a portable freezer (Alpicool ENX42, Foshan Alpicool Electrical Appliance Co., Ltd., Foshan city, China). All captured arthropods were taken to the laboratory, placed in Petri dishes over dry ice and examined using a Zeiss stereomicroscope (Carl Zeiss Digital Innovation GmbH. Germany) for taxonomic identification. The respective taxonomic levels—species, family, and order—and ecological function were determined based on a database [78]. When samples were too degraded or diagnostic morphological characters were hard to distinguish, identification was performed at the family level.

### 4.3. Community Parameters and Calculation Formulas

Number of individuals (*N*), species richness (*S*), Shannon–Wiener diversity index (*H′*), Evenness index (*J*) and Simpson’s dominant concentration (*C*) were used to analyze the structure and temporal dynamics of the NTA communities. *S* is the number of species within a defined region, which reflects the complexity and heterogeneity of the regional environment. *H′* and *C* are the ways to measure the diversity of species in a community; they can reflect changes in the populations of various species. *J* can be used as a measure of species dominance in a community, a measure of how close different species are to each other in number. The Berger–Parker dominance index (*D*) was calculated separately based on the different functions in the fields. *D* is an index reflecting community dominance and is the most sensitive to changes in community diversity. The similarity in species composition of NTA communities was analyzed using the Czekanowski Community Similarity Coefficient (*C_S_*). *C_S_* is used to compare the similarities and differences between biological community structures in different spaces. The formulas for calculation of the above indexes are as follow [79]:

$$H\prime =-\overset{s}{\underset{i=1}{\sum }}P_{i}\ln P_{i}$$

$$J=\frac{H\prime }{\ln S}$$

$$D=\frac{N_{\max}}{N}$$

$$D=\frac{N_{\max}}{N}$$

$$C=\overset{s}{\underset{i=1}{\sum }}{\left(\frac{n_{i}}{N}\right)}^{2}$$

$$C_{S}=\frac{2A}{a+b}$$

In the formulas: *S* is the species richness of the community; P_i_ is the ratio of the number of individuals of species *i* in the community to the total number of individuals in the community; n_i_ is the number of individuals of species i; *N* is the total number of individuals in the community; and *N*_max_ is the number of dominant species. When the species dominance *D* ≥ 0.1, it is a dominant species; when 0.01 ≤ *D* < 0.1, it is a common species; when *D* ≤ 0.01, it is a rare species [79]. *A* is the number of species shared by the two communities *a* and *b*, and *a* and *b* are the numbers of species in the corresponding communities *a* and *b*, respectively.

### 4.4. Statistical Analysis

All data are presented as mean ± standard error (SE), unless otherwise indicated. A Student’s *t*-test was used to compare the composition and community parameters of arthropods in SK12-5 (*Bt*) and LC303 (non-*Bt*) maize fields. Composition and characteristics of NTAs in the same maize fields at different growth stages were analyzed by one-way ANOVA followed by the Tukey HSD test. In addition, the composition of NTAs in the orders Araneae, Dermaptera, Diptera, Entomobryomorpha, Hemiptera, Hymenoptera and Thysanoptera in LC303 (non-*Bt*) maize fields, and the composition of NTAs in the orders of Araneae, Dermaptera, Entomobryomorpha, Hemiptera, Hymenoptera and Neuroptera in SK12-5 (*Bt*) maize fields were analyzed by the Mann–Whitney U-test because of the associated heterogeneity of variance. Differences were considered as significant at *p* < 0.05. These analyses were conducted using the SPSS Version 13.0 statistical software. Community structure was determined with NMDS (non-metric multidimensional scaling) ordinations based on Bray–Curtis dissimilarity. NMDS is a multivariate, nonlinear technique that ranks points such that distance in ordination space represents similarity among sample periods [80] (pp. 91–173). The correspondence of the ordination diagram to the similarity distances is described by a stress value, where 0 is a perfect fit. Furthermore, analysis of similarity (ANOSIM) was used to test if there was a statistical difference among the NTA communities, sample time and maize types [58,77]. This analysis was conducted with the vegan package in R (v.3.2.3; R Development Core Team).

## 5. Conclusions

This study collected NTAs in *Bt* and non-*Bt* maize fields during three main growing stages around maize flowering periods. By analyzing the compositions of NTAs in these three stages, the ecological niche change from domination by Hemiptera to Coleoptera was observed. Meanwhile, long-term and large-scale planting of *Bt* maize requires attention to the population dynamics of NTAs such as aphids and other pests to avoid pest outbreaks due to less pesticide use. The cultivation of *Bt* maize expressing Cry1Ab/2Aj protein did not show any negative impacts on the densities of non-target decomposers, herbivores, predators, parasitoids and pollinators. The compositions of decomposers, herbivores, predators, parasitoids and pollinators were similar in *Bt* and non-*Bt* maize fields. Taken together, results from our work support the view that planting of *Bt* maize producing Cry1Ab/2Aj toxins does not adversely affect populations of NTAs. Moreover, this study shows that the changes in the abundance and diversity of NTAs in maize fields are driven by time, and the Cry1Ab/2Aj toxin exposure plays a negligible, if any, role in the evolution of these NTA communities. Interactions between maize and NTAs occur over a wide range of timescales from hours to seasons and years and are mostly driven by temperature, insolation or rainfall. Hence, long-term and large-scale studies need to take into account a large variety of environmental parameters, including the effects of insecticide treatments on non-*Bt* crops, and it is also necessary to ensure the long-term efficacy of GE crops with reduced impact on the environment and agricultural ecosystems.

## Figures and Tables

**Table 1:** Species composition and the temporal dynamics of non-target arthropods (NTAs) in fields planted with transgenic *Bt* maize and non-*Bt* maize in Jilin, China.

Order	Family	Species	Investigation Date					
			BF ^1^		DF ^2^		AF ^3^	
			Non-*Bt* ^a^	*Bt* ^b^	Non-*Bt* ^a^	*Bt* ^b^	Non-*Bt* ^a^	*Bt* ^b^
Araneae	Agelenidae	Spider	++	++	++	++	++	++
	Araneida							
	Clubionidae							
	Hahniidae							
	Linyphiidae							
	Lycosidae							
	Pisauridae							
	Salticidae							
	Theridiidae							
	Thomisidae							
Coleoptera	Carabidae	/	++	++	+	+	+	++
	Chrysomelidae	/	+	++	++	+	+	+
		*Aulacophora femoralis* (Motschulsky, 1857)	+	+				+
		*Monolepta typographica* (Weise, 1915)	++	++	++	++	+	+
		*Pachnephorus lewisii* (Baly, 1878)	+	+				
	Coccinellidae	/			+		+	
		*Harmonia axyridis* (Pallas, 1773)	++	++	++	++	+++	+++
		*Propylaea japonica* (Thunberg, 1781)	++	++	++	++	+	+
		*Rodolia rufopilosa* (Mulsant, 1850)					+	
		*Scymnus hoffmanni* (Weise, 1879)		+		+		
	Curculionidae	/				+		+
	Elateridae	/						+
		*Aeoloderma agnata* (Candeze, 1873)				+		
		*Melanotus caudex* (Lewis, 1879)				+		
	Melolonthidae	*Holotrichia oblita* (Faldermann, 1835)		+		+		+
	Platypodidae	/				+		
	Rutelidae	*Popillia quadriguttata* (Fabricius, 1787)		+				
	Staphylinidae	*Paederus fuscipes* (Curtis, 1826)	+	+	+	++		+
	Tenebrionidae	*Opatrum subaratum* (Faldermann, 1835)	+	+	+	+		
		*Tribolium castaneum* (Herbst, 1797)	+	+	+	+	+	+
Dermaptera	Labiduridae	Labidura sp.	++	++	++	++	+	+
Diptera	Asilidae	/		+				
	Cecidomyiidae	*Aphidoletes aphidimyza* (Rondani, 1846)		+				
	Chironomidae	/	+					
	Culicidae	/	++	++	+	+	++	++
	Dolichopodidae	/		+				
	Drosophilidae	*Drosophila macquarti* (Wheeler, 1981)	+	+	+		++	++
	Empododae	/				+		
	Muscidae	/	++	++	++	++	++	++
		*Musca domestica* (Linnaeus, 1758)	+	+	+	+	++	+
	Sarcophagidae	*Sarcophaga melanura* (Meigen, 1826)	+	+	+			+
	Stratiomyidae	*Hermetia illucens* (Linnaeus, 1758)		+				
	Syrphidae	*Episyrphus balteatus* (De Geer, 1776)	+		+		++	++
		*Eupeodes corollae* (Fabricius, 1794)						+
		*Melanostoma scalare* (Fabricius, 1794)						+
		*Pipizella varipes* (Meigen, 1822)				+		+
		*Sphaerophoria menthastri* (Linnaeus, 1758)						+
	Tabanidae	/		+			+	++
		*Tabanus amaenus* (Walker, 1848)				+		
		*Tabanus signatipennis* (Portschinsky, 1887)		+				
		*Tabanus* sp.	+					+
	Tephritidae	/	++	++	+	+	+	+
Entomobryomorpha	Isotomidae	*Isotomidae* sp.	++	++	++	++	++	++
Ephemeroptera	Baetidae	/	+	+	+	+		
Hemiptera	Anthocoridae	*Orius strigicollis* (Poppius, 1915)	++	++	+	++	+	+
	Aphididae	/			++	++		++
		*Rhopalosiphum maidis* (Fitch, 1856)	+++	+++	+++	+++	+++	++
		*Rhopalosiphum padi* (Linnaeus, 1758)	+++	+++	+++	+++	++	++
	Cicadellidae	*Cicadella viridis* (Linnaeus, 1758)			+	+		
		*Macropsis notata* (Prohaska, 1923)		+				
		*Psammotettix striatus* (Linnaeus, 1758)	+	+				
	Cydnidae	*Adrisa magna* (Uhler, 1861)	+	+				
	Delphacidae	/						+
		*Laodelphax striatellus* (Fallén, 1826)	+	+	+	+	+	+
		*Sogatella furcifera* (Horváth, 1899)	+	+	+	+	+	+
		*Trigonotylus ruficornis* (Geoffroy in Fourcroy, 1785)	+	++	+	++	+	+
	Miridae	*Adelphocoris* sp.				+		+
		*Apolygus lucorum* (Meyer-Dur, 1843)			+			
		*Cyrtorhinus lividipennis* (Reuter, 1885)	+		+	+		
		*Lygus pratensis* (Linnaeus, 1758)			+			
	Nabidae	*Nabis stenoferus* (Hsiao, 1964)				+		+
Hymenoptera	Formicidae	*Lasius fuliginosus* (Latreille, 1798)	++	+++			++	++
	Apidae	*Apis cerana* (Fabricius, 1793)	+	+	+	+		
	Eumenidae	/					++	++
	Megachilidae	/		+			+	++
	Vespidae	/	+		+			
	Aphelinidae	Parasitic Wasp	++	++	++	++	+	+
	Braconidae							
	Eulophidae							
	Ichneumonidae							
	Pteromalidae							
	Scelionidae							
	Trichogrammatidae							
Neuroptera	Chrysopidae	*Chrysopa pallens* (Rambur, 1838)		+				
		*Chrysoperla nipponensis* (Okamoto, 1914)	+	+	++	++	++	++
	Hemerobiidae	/			+		+	+
		*Micromus timidus* (Hagen, 1853)			+		+	+
Orthoptera	Acrididae	/	+		+			
	Coenagrionidae	*Ischnura asiatica* (Brauer, 1865)						+
	Gryllidae	*Gryllidae* sp.			+			
Thysanoptera	Aeolothripidae	*Aeolothrips fasciatus* (Linnaeus, 1758)	+	+		+		
	Phlaeothripidae	*Gynaikothrips uzeli* (Zimmermann, 1900)		+				
		*Haplothrips aculeatus* (Fabricius, 1803)				+		
	Thripidae	*Anaphothrips obscurus* (Müller, 1776)		+	+	+		

“^1^” BF—Before Flowering stage; “^2^” DF—During Flowering stage; “^3^” AF—After Flowering stage; “^a^” represents the LC303 fields that planted the non-transgenic near isoline maize of SK12-5; “^b^” represents the SK12-5 fields that planted transgenic *Cry1Ab/2Aj* maize; “^+++^” denotes dominant species; “^++^” denotes common species; “^+^” denotes rare species.

**Table 2:** Comparison of community parameters for arthropods between fields planted with *Bt* maize or non-*Bt* maize. Values represent means ± SE, *n* = 7 replicates. The differences in the same maize fields among months were analyzed by a one-way ANOVA followed by the Tukey HSD test (all *p* > 0.05). The differences of the community parameters of arthropods between *Bt* and non-*Bt* maize fields were analyzed by a Student’s *t*-test (an asterisk denotes a significant difference, *p* < 0.05).

Parameter of Community	BF ^1^		DF ^2^		AF ^3^	
	Non-*Bt* ^a^	*Bt* ^b^	Non-*Bt* ^a^	*Bt* ^b^	Non-*Bt* ^a^	*Bt* ^b^
**Species richness (*S*)**	8.43 ± 3.14	11.71 ± 2.63	10.71 ± 3.60	11.00 ± 2.72	8.00 ± 1.41	10.86 ± 1.90
**Shannon-Wiener diversity index (*H′*)**	1.10 ± 0.24	1.50 ± 0.35	1.23 ± 0.26	1.53 ± 0.21	1.15 ± 0.18	1.38 ± 0.17
**Evenness index (*J*)**	0.63 ± 0.068	0.63 ± 0.096	0.60 ± 0.11	0.70 ± 0.082	0.58 ± 0.060	0.60 ± 0.050
**Simpson’s dominant concentration (*C*)**	0.47 ± 0.062	0.45 ± 0.15*	0.45 ± 0.11	0.31 ± 0.064	0.48 ± 0.071	0.41 ± 0.057
**Czekanowski Community Similarity Coefficient (*C_S_*)**	0.73		0.67		0.7	

“^1^” BF—Before Flowering stage; “^2^” DF—During Flowering stage; “^3^” AF—After Flowering stage; “^a^” represents the LC303 fields that were planted with the non-transgenic near isoline maize of SK12-5; “^b^” represents the SK12-5 fields that were planted with transgenic *Cry1Ab/2Aj* maize.

## Data Availability

Due to privacy the data presented in this study are available on request from the corresponding author.
